# Clinical Cases

**Published:** 1887-08

**Authors:** J. T. Kent

**Affiliations:** St. Louis


### CLINICAL CASES.

PROFESSOR J. T. KENT, M. D., ST. LOUIS.

*Ulcerated Throat.*—Lady, thirty-four years old, mother of two children. Face marks much sickness, though flushed. Letter states: "I have always been troubled most with left side of my throat, but at present it is the right. A small lump will come and then enlarge until it reaches the tonsil. Then ulcers will come and fill both sides. The roof becomes very red, and there is dryness and choking. Dry choking compels coughing; difficult swallowing." I further learned that this sore throat with ulceration has been coming just before menstruating for several years. It commences on one side and goes to the other. There has always been great swelling of the outside, sometimes the whole neck. The ulcers do not disappear until after the flow ceases; then a gradual subsiding; scarcely more than ten days of freedom from suffering. Leucorrhœal discharge, white mucus before menses. She got Mag. carb.$^{45m}$ (Fincke), one dose, at the close of menstrual *nisus*. She has never had a recurrence of the trouble nor any sickness in its place. She has remained free now over two years.

*Pulsatilla.*—Mrs. P., aged forty-two, has been a most miserable sufferer for several years, trying to have comfort through allopathy. Symptoms: Pain in the heels like the pricking of tacks or nails; hot flushes, followed by chilliness; menstrual flow black and clotted; puts feet out of bed to cool them, they burn so; she must put her shoes on before she can walk, "heels ache so;" vertigo mostly before menses; she has been deaf since childhood, from scarlet-fever; constipation, character not ascertained; open air is grateful, craves open air; warm room is oppressive, she suffocates and must go out into the air; church is oppressive; watery discharge from eyes and nose; purplish appearance of the skin of the heel; sprained feeling in the ankles, weak ankles.

May 23d.—Puls.$^{51m}$ (F.), one dose, and plenty of Sac. lac.

June 30th.—Puls.$^{cm}$ (F.) and Sac. lac. She needed no medicine until April 13th, the next year, when she consulted me with the following symptoms: Rattling cough; loses her urine when coughing; feels stopped up in a warm room; menses every two weeks, profuse, dark, offensive; urine offensive, strong; sharp pains in rectum; toe joints very sore; hot flushes; limbs tire easily when walking. Puls.$^{cm}$ one dose.

April 26th.—Felt so much heat in vulva that she was compelled to apply a cold cloth; no appetite; sleepless; burning heat all over body; throws covers all off the bed; "I feel no two days alike," "I am so fidgety." She got more Sac. lac.

May 3d.—Says she is well; plenty of Sac. lac. June 20th.—Loses her urine when coughing. July 10th.—The same symptom continues to bother her. Puls.$^{cm}$ (F.) finished the cure and she remains well.

